# In Vitro Mechanistic Studies of a Standardized Sustainable Grape Seed Extract for Potential Application as a Mood-Modulating and Cognition-Enhancing Supplement

**DOI:** 10.3390/nu16203459

**Published:** 2024-10-12

**Authors:** Gozde Hasbal-Celikok, Mehtap Kara, Marta Sánchez, Claudia Owsianik, Pilar Gómez-Serranillos, Tugba Yilmaz-Ozden, Ezgi Öztaş, Özge Sultan Zengin, Gul Ozhan, Nazli Arda, Merve Tunc, Sumeyye Sahin, Areaba Shafiq, Ayesha Kanwal, Hunaiba I. Ujjan, Fazle Rabbani, Giovanna Petrangolini, Amjad Khan

**Affiliations:** 1 Department of Biochemistry, Faculty of Pharmacy, Istanbul University, Istanbul 34452, Türkiye; gozde.hasbal@istanbul.edu.tr (G.H.-C.); tugbay@istanbul.edu.tr (T.Y.-O.); 2 Department of Pharmaceutical Toxicology, Faculty of Pharmacy, Istanbul University, Istanbul 34452, Türkiye; mehtap.kara@istanbul.edu.tr (M.K.); ezgi.oztas@istanbul.edu.tr (E.Ö.); ozgesultanzengin@gmail.com (Ö.S.Z.); gulozhan@istanbul.edu.tr (G.O.); 3 Department of Pharmacology, Pharmacognosy and Botany, Faculty of Pharmacy, Complutense University of Madrid, 28040 Madrid, Spain; martas15@ucm.es (M.S.); claudiow@ucm.es (C.O.); pserra@ucm.es (P.G.-S.); 4 Department of Molecular Biology and Genetics, Faculty of Science, Istanbul University, Istanbul 34452, Türkiye; narda@istanbul.edu.tr (N.A.); merve.tunc.2018@ogr.iu.edu.tr (M.T.); 5 Department of Food Engineering, Ordu University, Ordu 52200, Türkiye; gmsumeyyesahin@gmail.com; 6 Department of Psychiatry, Lady Reading Hospital, Peshawar 25000, Pakistan; areabashafiq@gmail.com (A.S.); ayeshazaib997@gmail.com (A.K.); fazalerabbani007@gmail.com (F.R.); 7 Department of Pathology, Liaquat University of Health Sciences, Jamshoro 76090, Pakistan; hunaibaujjan@gmail.com; 8 Medical Department, Indena SpA, 20139 Milan, Italy; 9 Department of Oncology, University of Oxford, Oxford OX3 7DQ, UK; 10 Department of Biochemistry, Liaquat University of Medical & Health Sciences, Jamshoro 76090, Pakistan

**Keywords:** Enovita^®^, GSEe, grape seed extract, low mood, cognitive function, sustainability, circular economy

## Abstract

Background: Grape seed extract (GSE) from *Vitis vinifera* L. is rich in polyphenols and oligomeric proanthocyanidin complexes (OPCs), and it has shown potential benefits in managing low mood and cognitive function. In this study, we investigated the potential bioactivities of Enovita^®^, a standardized GSE extract (GSEe herein) rich in OPCs, in key mechanistic pathways related to low mood conditions and cognitive function. Methods: In vitro assays were conducted to assess GSEe’s inhibitory effects on γ-aminobutyric acid transaminase (GABA-T) and monoamine oxidase A (MAO-A), its binding affinity to the GABA site of GABA-A receptors, and its effects on acetylcholinesterase (AChE). Its neuroprotective effects on human SH-SY5Y neuroblastoma cells under oxidative stress (induced by H_2_O_2_) were assessed using MTT and LDH release assays. Its antioxidant activities were evaluated using DPPH, ABTS, FRAP, ORAC, HORAC, total phenolic content, and TAS assays. Its cytotoxicity was also evaluated. Results: GSEe showed significant GABA-T inhibitory activity. It also exhibited MAO-A and AChE inhibition, along with moderate binding affinity to the GABA-A receptor. In neuroprotective assays, GSEe provided significant protection to SH-SY5Y cells against oxidative stress. GSEe demonstrated robust antioxidant activity in all assays, including scavenging of DPPH and ABTS radicals, high ferric-reducing power, high polyphenolic contents, and a substantial total antioxidant capacity. Conclusions: GSEe exhibits promising bioactivities, highlighting its potential as a supplement for modulating mood and enhancing cognitive function. Overall, the promising results from these in vitro studies provide a strong foundation for the continued exploration and development of GSEe as a viable natural supplement for enhancing mental health and cognitive function.

## 1. Introduction

Grape seed extract (GSE), derived from the seeds of *Vitis vinifera* L., has garnered significant interest in recent years due to its potent antioxidant properties and potential benefits in the management of low mood conditions and cognitive function. Rich in polyphenols [1], particularly oligomeric proanthocyanidin complexes (OPCs), GSE has been extensively studied for its role in improving mental health and cognitive performance through various pharmacological mechanisms [2,3,4,5,6,7,8,9]. Moreover, several human clinical studies have demonstrated the efficacy of GSE in enhancing cognitive function and alleviating symptoms of low mood, further substantiating its potential health benefits [10,11,12,13,14,15,16].

Conventional treatments, including antidepressant medications and cognitive enhancers, often come with limitations such as adverse side effects, the development of tolerance, delayed onset of therapeutic effects, and inconsistent efficacy [17,18,19]. Subjects may experience weight gain, sexual dysfunction, and difficulty discontinuing the medication due to withdrawal symptoms or relapse of their condition [17,18,19]. These challenges underscore the need for alternative or complementary approaches to manage low mood conditions and cognitive decline.

GSE, with its high concentration of antioxidants [20], has been suggested as a potential natural support for managing low mood and enhancing cognitive function. The bioactive compounds in GSE, particularly OPCs, are known to exert neuroprotective effects [21], modulate neurotransmitter systems, and reduce oxidative stress and inflammation [22,23,24,25], which are critical factors in mood regulation and cognitive health. Additionally, GSE has demonstrated favorable safety and tolerability profiles in clinical studies [10,11,12,13,14,15,16], making it a promising alternative or complementary approach for managing low mood and related conditions. The extract utilized in the present research was a highly standardized grape seed extract (Enovita^®^, referred to as GSEe herein) from *Vitis vinifera* L. rich in polyphenols, mainly composed of oligomeric proanthocyanidins, and also containing monomeric procyanidins (catechin and epicatechin).

The peculiar characteristic of GSEe is the high concern regarding sustainability and how the circular economy can start a virtuous cycle. This natural-origin extract is made by upcycling natural raw materials that are usually discarded; thus, waste is given a new life and put through an eco-friendly extraction process implemented to save resources, minimize emissions, and exclusively use water as a solvent. Residual materials are transformed into food oil, fertilizers, and energy [16,26].

This study aims to address a critical gap by investigating the potential bioactivities of a standardized grape seed extract, named GSEe, in various key mechanistic pathways implicated in low mood conditions and cognitive function. Through a series of in vitro assays, this research seeks to elucidate the molecular mechanisms underpinning these effects and to provide a comprehensive understanding of how GSEe supplementation could serve as an effective natural strategy for managing low mood conditions and cognitive decline.

## 2. Materials and Methods

### 2.1. Assays

All assays were conducted in triplicate using three freshly prepared samples. They either followed a reported protocol or utilized commercially available assay kits. A positive control was included in all assays. Unless specified otherwise, all chemicals used were commercially purchased, and solutions were prepared in an aqueous buffer.

### 2.2. Grape Seed Extract

The standardized food-grade grape seed extract GSEe (Enovita^®^) was provided by Indena SpA (Milan, Italy). GSEe is an extract rich in OPCs, derived exclusively from recovered grape seeds used in white wine production close to the processing site. The extraction process is carried out using only water, without the use of other less environmentally friendly solvents, so the GSEe production process meets sustainability criteria. GSEe is produced under HACCP conditions in a GMP- and ISO 14001-certified [27] facility that is also equipped with photovoltaic panels, which allow for conscious and sustainable energy use. All this ensures full traceability from grape harvest to the finished product and a strong commitment to fighting climate change. With water as the extraction solvent, GSEe is standardized to contain ≥95.0% OPCs (by spectrophotometry) and 5.0–15.0% flavane monomers (catechin and epicatechin, by HPLC) [16,26].

### 2.3. Human SH-SY5Y Cell Culture

The human SH-SY5Y neuroblastoma cell line was obtained from the American Type Culture Collection (ATCC, # CRL-2266™) [28]. The cells were cultured in DMEM/F-12 medium supplemented with 10% fetal bovine serum (FBS) and 100 U/mL penicillin–streptomycin. Incubation was carried out at 37 °C in a humidified atmosphere containing 5% CO_2_. Cells were passaged every 3 to 4 days upon reaching confluence. Cells were seeded at densities depending on the specific requirements of each experiment. The extract was applied to the cell cultures for a 24 h exposure period.

### 2.4. Neurotransmitter-Modulating Target Assays

#### 2.4.1. γ-Aminobutyrate Transaminase (GABA-T) Inhibition Assay

This assay was conducted using a commercial kit according to the manufacturer’s protocol (BRSC, University at Buffalo, Buffalo, NY, USA, # E-134). GSEe (1 mg) was dissolved in 1 mL of boiled distilled water (dH_2_O) and agitated at room temperature for 30 min to prepare a 1 mg/mL stock solution. This stock was then serially diluted with dH_2_O. The GABA-T enzyme source was rat brain extract with a protein concentration of 5 mg/mL. Each dilution of GSEe (10 μL) was added to a 96-well plate, with control (C) and reaction (R) wells set up in triplicate. Rat brain GABA-T extract (25 μL, containing 125 μg of protein) was added to each well and incubated at room temperature for 10 min. To the respective wells, 40 μL of dH_2_O (for control wells) or GABA-T substrate (for reaction wells) was added, followed by incubation at 37 °C for 1 h. Subsequently, 50 μL of TA assay solution was added to each well and incubated again at 37 °C for 1 h. The optical density (OD) was measured at 492 nm (OD492) using a plate reader. The GABA-T enzyme activity was calculated by subtracting the OD of the control wells (ODC) from the OD of the reaction wells (ODR). A linear regression analysis was performed to determine the concentration-dependent inhibitory effect of GSEe on GABA-T activity, and IC_50_ values were calculated from the dose–response curves.

#### 2.4.2. γ-Aminobutyric Acid Type A (GABA-A) Receptor-Binding Assay

The binding assay of GSEe to the GABA site of GABA-A receptor was conducted following Heaulme et al.’s method with modifications [29]. Rat brain tissue was used as the receptor source. [3H]-Muscimol (6 nM) (specific activity: 25.2 Ci/mmol, RC Tritec AG, Teufen, Switzerland, # RCTT0324) served as the radioligand, and SR95531 (31.6 μM) (Sigma-Aldrich Chemie GmbH, Taufkirchen, Germany, # BN0507) was used to determine non-specific binding. Incubation was performed for 30 min at 4 °C, and scintillation counting quantified the binding activity. Brain tissue, stored at −80 °C, was homogenized in 10 mM Tris-HCl pH 7.4 with 1 mM EDTA, then centrifuged at 25,000× *g* for 15 min. This process was repeated twice, and the final pellet was resuspended in 50 mM Tris-HCl pH 7.4, 4 mM MgCl_2_, and 1 mM EDTA, then stored at −80 °C.

GSEe was solubilized in H_2_O Milli-Q^®^, and further dilutions were prepared. Membranes were thawed, centrifuged at 16,000× *g* for 10 min at 4 °C, and resuspended in assay buffer (50 mM Tris-Citrate, pH 7.1). After a 30 min incubation at 37 °C and subsequent centrifugation, the membranes were resuspended and incubated in assay buffer at 4 °C for 30 min. The assay was terminated by transferring samples onto GF/C filter plates presoaked with 0.05% Pluronic F127 in 50 mM Tris-HCl pH 7.4. The filters were washed with ice-cold 50 mM Tris-HCl pH 7.4, and the bound radioactivity was measured using a microplate reader (Microbeta, Wallac Oy, Turku, Finland). Data for reference compounds are presented as the total bound radioactivity (in cpm). GSEe binding is shown as specific radioligand binding to the receptor, defined as the difference between total and non-specific binding determined in the presence of reference compounds. IC_50_ values were determined by a non-linear regression analysis of competition curves using the “sigmoidal dose–response” algorithm (GraphPad Prism, San Diego, CA, USA). For Hill-defined curves or algorithm-generated minima below non-specific binding, IC_50_ values were graphically extrapolated.

#### 2.4.3. Monoamine Oxidase A (MAO-A) Inhibition Assay

The MAO-A inhibition assay was conducted using the MAO-A Inhibitor Screening Kit (OxiSelect™, CellBiolabs, San Diego, CA, USA, # STA-324), following the manufacturer’s protocol. Briefly, 50 μL amounts of the kit standard, control, and GSEe sample solution at various concentrations were added to individual wells of a microtiter plate. Subsequently, 50 μL of the assay working solution was added to each well. The contents of the wells were mixed thoroughly, and the microplate was incubated at room temperature for 60 min in the dark. After incubation, the absorbance was measured using a microplate reader (Biotek, Epoch, VT, USA) at a wavelength range of 540–570 nm. The results are expressed as the relative inhibition (%).

### 2.5. Acetylcholinesterase (AChE) Inhibition Assay

This assay was carried out based on a previously reported method [30]. Briefly, 20 μL of GSEe at various concentrations was combined with 180 μL of Ellman’s reagent solution. This solution contained 318 mM DTNB (Sigma # D8130) and 955 mM ATChI (Sigma # A5751) in 0.1 M phosphate buffer at pH 7.5. Subsequently, 50 μL of AChE solution (Sigma # C3389; 0.5 U/mL) was added to the mixture. The reaction’s progress was monitored by measuring the absorbance change at 412 nm over a 10 min period. Control experiments were conducted by substituting the extract with 20 μL of the solvent. Galantamine (Sigma # G1660) was used as a standard reference inhibitor for AChE. The percentage of inhibition of AChE activity was calculated using the following formula:

$$Enzyme\;inhibitory\;activity\;\left(\%\right)=\left(1-\left(\frac{Absorbance\;change\;of\;sample\;at\;412\;nm}{Absorbance\;change\;of\;control\;at\;412\;nm}\right)\right)\times 100$$

IC_50_ values were calculated by linear regression analysis from dose–response curves.

### 2.6. Protection of SH-SY5Y Cells against H_2_O_2_-Induced Oxidative Stress: Assays

#### 2.6.1. MTT (3-[4,5-Dimethylthiazol-2-yl]-2,5-diphenyl Tetrazolium Bromide) Assay

This assay was carried out according to a previously reported method [31]. Briefly, SH-SY5Y cells (1 × 10^4^ cells/well) were incubated in a 96-well plate for 24 h to allow for attachment; then, the medium was refreshed and the cells were co-treated with 150 µM H_2_O_2_ and various concentrations of GSEe and incubated for an additional 24 h. Subsequently, MTT solution (5 mg/mL) was added to each well, and the cells were incubated for 3 h at 37 °C. The reduction of MTT to formazan, indicative of metabolic activity, was measured by determining the absorbance at 590 nm using a Biotek Epoch™ microplate spectrophotometer (Agilent, Santa Clara, CA, USA).

#### 2.6.2. Lactate Dehydrogenase (LDH) Release Assay

This assay was performed based on a previously reported method [32]. Briefly, SH-SY5Y cells (1 × 10^4^ cells/well) were seeded in a 96-well plate and incubated for 24 h. After the incubation period, the medium was refreshed, and cells were then co-treated with 150 µM H_2_O_2_ and various concentrations of GSEe solution for an additional 24 h. LDH release, a marker of membrane damage, was quantified using an LDH cytotoxicity assay kit (Roche Diagnostics, Instanbul, Turkey, # 11644793001) according to the manufacturer’s protocol. Triton X-100 (1%) served as a positive control. The optical density was measured at 495 nm using a microplate reader, and the percentage of LDH release was calculated based on a linear regression curve.

### 2.7. Cell-Free Antioxidant Activity Assays

#### 2.7.1. DPPH (2,2-Diphenyl-1-picrylhydrazyl) Radical Scavenging Activity Assay

The DPPH radical scavenging activity assay was performed based on a modified version of Brand-Williams et al.’s method [30,33]. Briefly, 10 μL of GSEe solution at various concentrations was mixed with 240 μL of 0.1 mM DPPH radical solution. The mixture was incubated at room temperature in the dark for 30 min. The absorbance decrease, indicating the reduction of DPPH radicals, was then measured at 517 nm using a methanol blank. Quercetin served as the standard antioxidant. A control was prepared using the solvent instead of the extract. The DPPH radical scavenging activity (%) was calculated using the following equation:

$$\mathrm{DPPH}\;\mathrm{radical}\;\mathrm{scavenging}\;activity\;\left(\%\right)=1-\left(\frac{Absorbance\;of\;\mathrm{the}\;\mathrm{extract}at\;517\;nm}{Absorbance\;of\;\mathrm{the}\;\mathrm{control}\;at\;517\;nm}\right)\times 100$$

The half-maximal effective concentration (EC_50_) values of GSEe were derived from the percentage of DPPH radical inhibition.

#### 2.7.2. ABTS (2,2′-Azinobis-(3-ethylbenzothiazoline-6-sulfonic Acid)) Radical Scavenging Activity Assay

The ABTS radical scavenging activity of GSEe was assessed using a modified method from Re et al. [34]. Briefly, an ABTS stock solution (7 mM) was prepared by reacting ABTS with potassium persulfate (final concentration 2.45 mM) and incubating it in the dark for 16 h. This stock was diluted with ethanol to an absorbance of 0.70 ± 0.02 at 734 nm. A 5 μL sample of GSEe solution at various concentrations was mixed with 245 μL of the ABTS working solution. After 6 min, the decolorization was measured at 734 nm using a spectrophotometer (Biotek, Epoch, VT, USA). Quercetin served as the standard, and a control was prepared using the solvent.

The ABTS radical scavenging activity was calculated as follows:

$$\mathrm{ABTS}\;\mathrm{radical}\;\mathrm{scavenging}\;activity\;\left(\%\right)=\left(1-\frac{Absorbance\;of\;\mathrm{the}\;\mathrm{extract}at\;734\;nm}{Absorbance\;of\;\mathrm{the}\;\mathrm{control}\;at\;734\;nm}\right)\times 100$$

The EC_50_ values were derived from the percentage of ABTS radical inhibition.

#### 2.7.3. Ferric-Reducing Antioxidant Power (FRAP) Assay

The antioxidant activity of GSEe was measured using the ferric-reducing antioxidant power assay as described by Avan and colleagues, with some modifications [35]. This method involves the reduction of the ferric tripyridyl triazine (Fe[III]-TPTZ) complex to its colored ferrous form (Fe[II]-TPTZ) in the presence of antioxidants. FRAP reagent (TPTZ in HCl, FeCl₃, and acetate buffer, pH 3.6), freshly prepared and warmed to 37 °C, was used. Aliquots of 110 µL of the samples were mixed with 900 µL of the FRAP reagent and incubated at 37 °C for 10 min. After a 10 min incubation, the absorbance was measured at 595 nm using a SPECTROstar™ Nano Microplate Reader (BMG Labtech, Ortenberg, Germany). A calibration curve was constructed using five concentrations of FeSO_4_·7H_2_O (1, 0.8, 0.4, 0.1, 0.05 μmol). The results are expressed as the µmol equivalents of Fe (II) per gram of the sample solution.

#### 2.7.4. Oxygen Radical Antioxidant Capacity (ORAC) Assay

The ORAC assay was performed using a commercial kit, following the manufacturer’s protocol (BQC, Asturias, Spain # KF01004). Briefly, 15 µL of each standard and GSEe solution were added to a 96-well plate, followed by 90 µL of reagent B. After a 15 min incubation at 37 °C, 45 µL of reagent C was added to each well. The absorbance (fluorescence) was measured to assess the antioxidant activity, reflecting the ability of the sample to inhibit the reduction in fluorescence caused by the oxidative degradation of a fluorescent probe, using a fluorescent plate reader at excitation/emission wavelengths of 485 nm/528–538 nm (FLx800, BioTek (Agilent, Santa Clara, CA, USA) [36].

#### 2.7.5. Hydroxyl Radical Antioxidant Capacity (HORAC) Assay

This assay was performed using a commercial kit (MyBioSource, Inc., San Diego CA, USA, # MBS48047), following the manufacturer’s protocol. GSEe was diluted with methanol/water (70:30) and mixed with fluorescein. This mixture was added to all wells, including those with the samples and standards. The plate was incubated for 30 min at 37 °C, then hydroxyl radicals and Fenton reagent were added. The plate was shaken and read every minute for 45 min using an excitation wavelength of 488 nm and an emission wavelength of 515 nm. The results were compared to a standard curve of gallic acid to determine the gallic acid equivalents (GAE) per milligram of sample.

#### 2.7.6. Total Phenolic Content Assay

The total phenolic content (TPC) in GSEe was assessed using the Folin–Ciocalteau method [37,38,39]. Briefly, a reaction mixture of 10 µL GSEe solution (1 mg/mL), 200 µL Folin–Ciocalteau reagent, and 90 µL 7% sodium carbonate was prepared. After a 5 min incubation, the absorbance was measured at 595 nm using a SPECTROstar™ Nano Microplate Reader (BMG Labtech, Ortenberg, Germany) at 0 and 40 min (37 °C, in darkness) [40]. TROLOX (6-hydroxy-2,5,7,8-tetramethylchroman-2-carboxylic acid) was used as a standard reference. The TPC was calculated from a gallic acid standard curve and expressed as micrograms of GAE per mg of extract, with the results averaged from triplicate assays.

#### 2.7.7. Total Antioxidant Status (TAS) Assay

The TAS assay was performed using a commercial kit, following the manufacturer’s protocol (Elabscience™, Houston, TX, USA, # E-BC-K801-M). GSEe samples were diluted in 60% ethanol and mixed with the buffer solution in wells. An initial absorbance measurement (A1) was taken. Subsequently, a chromogenic solution containing oxidized ABTS was added, and the mixture was incubated for 5 min at 37 °C. A second absorbance measurement (A2) was recorded at 660 nm. The results were analyzed and compared to a Trolox standard, yielding the total antioxidant capacity in mmol of Trolox equivalents per kilogram of wet weight.

### 2.8. Cytotoxicity Assay

The cytotoxic effect of GSEe on SH-SY5Y cells, as indicated by a reduction in cell viability and metabolic activity, was assessed using the MTT assay as described in Section 2.6.1. Briefly, SH-SY5Y cells (1 × 10^4^ cells/well) were seeded into a 96-well plate and incubated for 24 h to allow for attachment. Afterward, the medium was refreshed, and the cells were treated with various concentrations of GSEe solution, followed by an additional 24 h incubation period. Subsequently, an MTT solution (5 mg/mL) was added to each well, and the cells were incubated for 3 h at 37 °C. The reduction of MTT to formazan, indicative of metabolic activity, was measured by determining the absorbance at 590 nm using a spectrophotometer (Biotek, Epoch, VT, USA).

### 2.9. Statistical Analysis

Data are expressed as the mean ± standard deviation (SD). Statistical analyses were performed with one-way ANOVA and post hoc Dunnett’s *t*-test using SPSS v.20 (IBM SPSS Inc., New York, NY, USA). A *p*-value of <0.05 was considered to indicate a statistically significant difference.

## 3. Results

### 3.1. GSEe’s Inhibitory Effect on GABA-T

**Figure 1:**
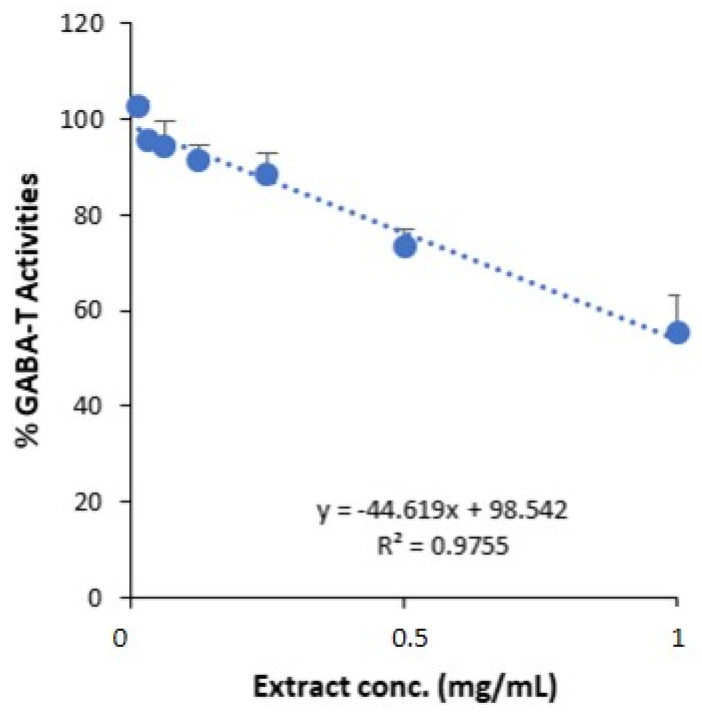
GABA-T inhibition assay of GSEe. The plot shows the percentage of GABA-T activity at various concentrations of GSEe, IC_50_ = 1.00 ± 0.20 mg/mL. A linear regression analysis was performed to determine the concentration-dependent inhibitory effect of GSEe on GABA-T activity. The error bars represent the standard deviation from the mean of the measurements of three freshly prepared samples.

The GABA-T inhibition assay for GSEe was conducted across a concentration range of 0.015 mg/mL to 1 mg/mL of GSEe and revealed a concentration-dependent decrease in GABA-T activity (Figure 1). The IC_50_ value was determined as 1 ± 0.20 mg/mL. The linear regression analysis showed a high correlation (R^2^ = 0.9755) for the equation y = −44.619x + 98.542, suggesting an inhibitory effect of GSEe on GABA-T activity.

### 3.2. GSEe’s GABA-A Receptor-Binding Activity

**Figure 2:**
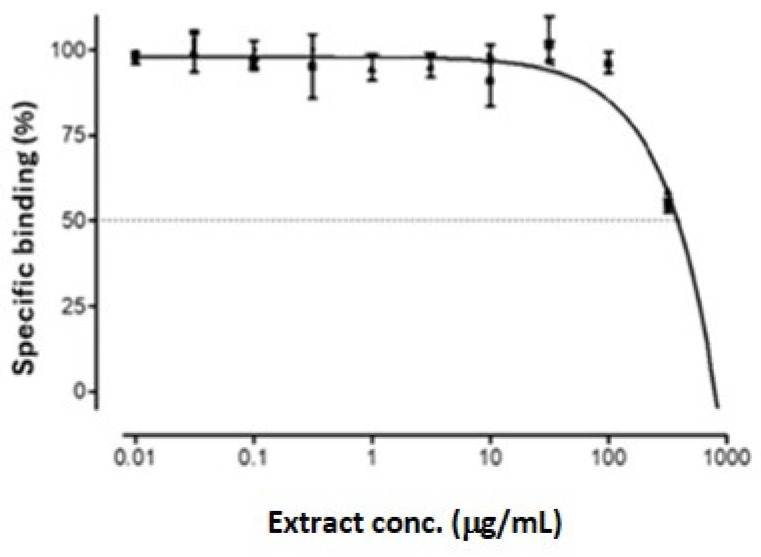
Competitive binding of GSEe at the GABA site of rat GABA-A receptors, IC_50_ = 394.5 μg/mL. The error bars represent the standard deviation from the mean of the measurements of three freshly prepared samples.

The specific binding of GSEe to the GABA site of the GABA-A receptor was assessed across a range of extract concentrations (0.01 µg/mL to 1000 µg/mL) and demonstrated an IC_50_ value of 394.5 ± 111 µg/mL (Figure 2). These results suggest that GSEe has a moderate affinity for the GABA-A receptor.

### 3.3. GSEe’s MAO-A Inhibitory Effect

**Figure 3:**
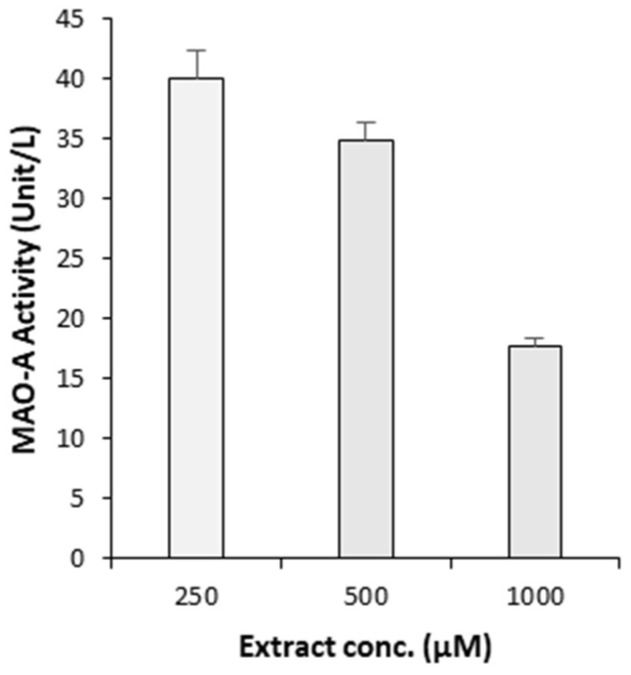
MAO-A enzyme inhibition assay. The plot shows the effect of various concentrations of GSEe on the activity of the MAO-A enzyme. The error bars represent the standard deviation from the mean of the measurements of three freshly prepared samples.

The MAO-A enzyme inhibition assay results show that GSEe significantly reduced MAO-A activity in a concentration-dependent manner across the tested range (250 µg/mL to 1000 µg/mL) (Figure 3), indicating its potential as an effective MAO-A inhibitor.

### 3.4. GSEe’s AChE Inhibitory Effect

**Figure 4:**
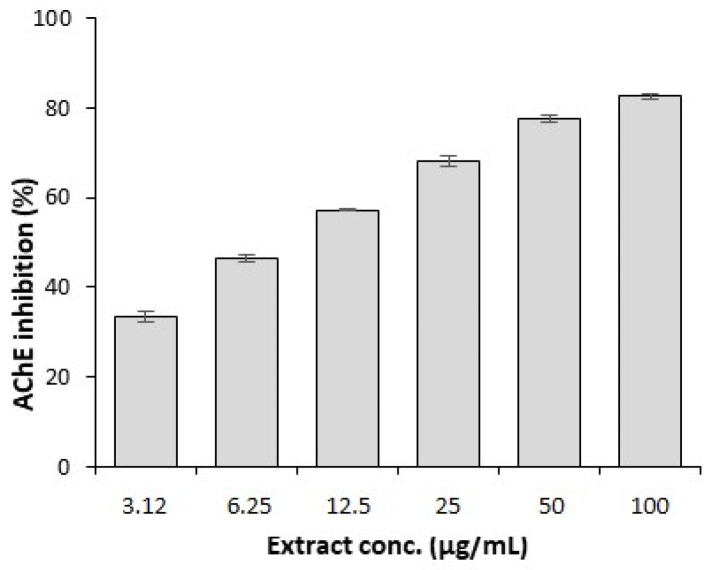
AChE enzyme inhibition assay. The plot shows the effect of various concentrations of GSEe on the activity of the AChE enzyme, IC_50_ = 11.44 ± 0.83 µg/mL. The error bars represent the standard deviation from the mean of the measurements of three freshly prepared samples.

The AChE enzyme inhibition assay results demonstrate that GSEe significantly decreased AChE activity in a concentration-dependent manner across the tested range (3.12 µg/mL to 100 µg/mL) (Figure 4), highlighting its potential as a potent AChE inhibitor. The IC_50_ value was obtained as 11.442 ± 0.827 µg/mL.

### 3.5. GSEe’s Neuroprotective Effect on SH-SY5Y Cells against H_2_O_2_-Induced Oxidative Stress

**Figure 5:**
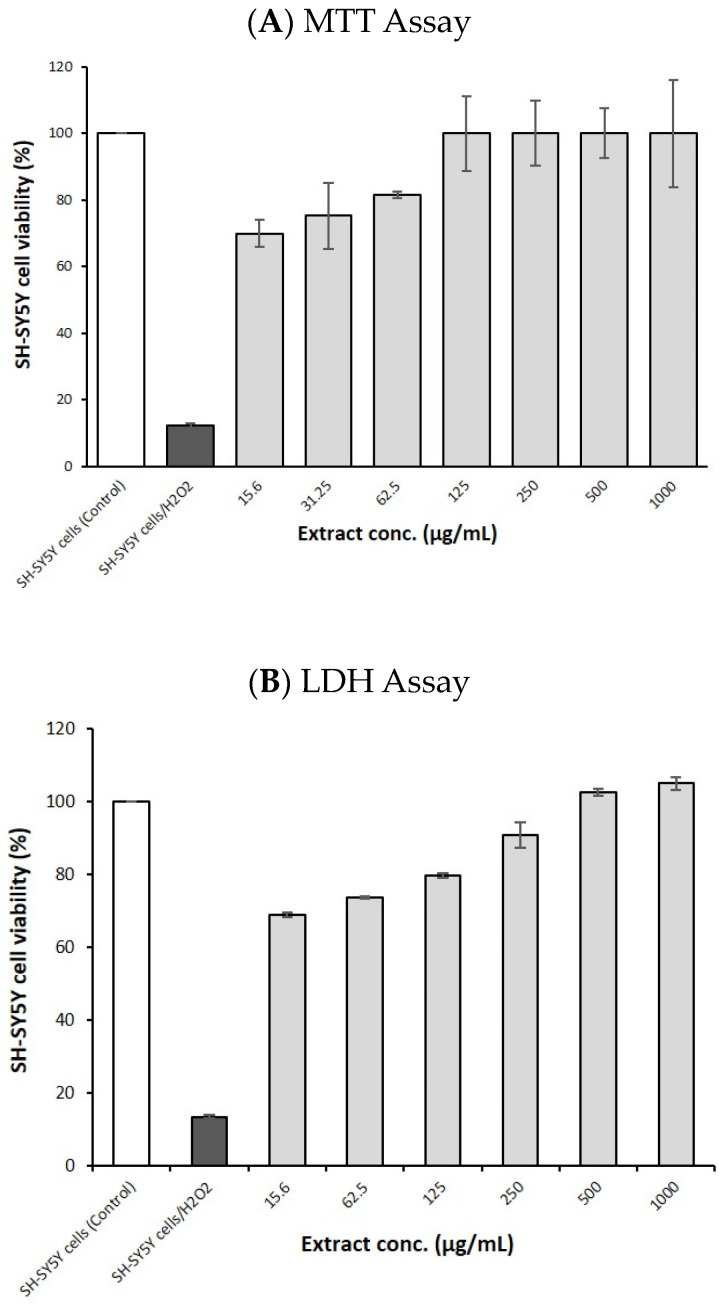
The neuroprotective effect of GSEe on SH-SY5Y cells under oxidative stress induced by H_2_O_2_. The plots show the effects of various concentrations of GSEe on SH-SY5Y cell viability. (**A**) MTT assay. (**B**) LDH assay. The error bars represent the standard deviation from the mean of the measurements of three freshly prepared samples.

In the MTT assay (Figure 5A), the treatment of SH-SY5Y cells under oxidative stress (induced by H_2_O_2_) with GSEe at various concentrations ranging from 15.6 µg/mL to 1000 µg/mL significantly improved cell viability in a dose-dependent manner, indicating the extract’s protective effect against oxidative stress. The LDH release assay also revealed similar results (Figure 5B), demonstrating that GSEe reduces cell damage and promotes cell survival under oxidative stress conditions.

### 3.6. GSEe’s Antioxidant Effect in Cell-Free Assays

**Figure 6:**
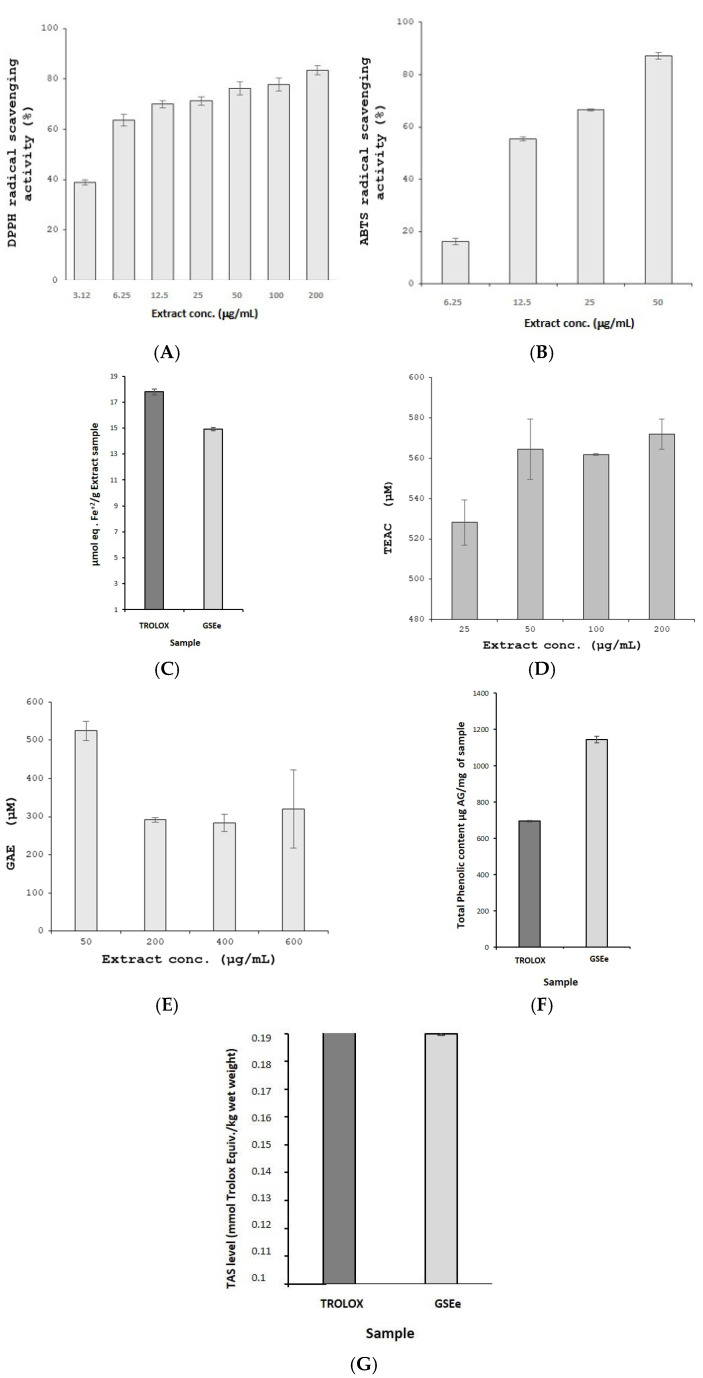
The antioxidant effects of GSEe, or Enovita^®^, in various cell-free assays. (**A**) DPPH radical scavenging assay, EC50 5.81 ± 0.11 µg/mL. (**B**) ABTS radical scavenging assay, EC50 11.91 ± 0.15 µg/mL. (**C**) FRAP assay. (**D**) ORAC assay. (**E**) HORAC assay. (**F**) Total phenolic content (TPC) Folin–Ciocalteau assay. (**G**) TAS assay. The error bars represent the standard deviation from the mean of the measurements of three freshly prepared samples. TEAC: Trolox Equivalent Antioxidant Capacity; GAE: gallic acid equivalents; GA: gallic acid.

GSEe demonstrated significant antioxidant activity across various cell-free assays, showing its robust potential. The DPPH radical scavenging assay exhibited a dose-dependent free radical scavenging effect within the concentration range of 3.12 to 200 µg/mL, indicating potent antioxidant properties (Figure 6A). The ABTS assay also confirmed GSEe’s strong antioxidant capacity through a concentration-dependent increase (Figure 6B). The FRAP assay further highlighted its substantial ferric-reducing power (Figure 6C). GSEe’s ability to neutralize peroxyl and hydroxyl radicals was underscored by the ORAC (Figure 6D) and HORAC (Figure 6E) assays, respectively, demonstrating comprehensive antioxidant efficacy.

Moreover, the TPC assay revealed significantly higher phenolic levels in GSEe compared to Trolox (Figure 6F), emphasizing its potent antioxidant capacity, as phenolic compounds are well known for their strong antioxidant properties. The TAS assay also reflected GSEe’s overall antioxidant status (Figure 6G), further validating its efficacy. Collectively, these findings suggest that GSEe possesses robust antioxidant potential, which may offer protective benefits for neuronal cells under oxidative stress conditions.

### 3.7. GSEe Cytotoxicity Assay

The MTT cytotoxicity assay results demonstrate that GSEe did not exhibit significant cytotoxic effects on SH-SY5Y cells across a range of concentrations (62.5 to 1000 µg/mL) (Figure 5A). The cell viability remained high and comparable to that of the control group (only culture medium), indicating that GSEe maintains cell viability even at higher concentrations.

## 4. Discussion

This study provides comprehensive insights into the potential of GSEe, or Enovita^®^, a standardized sustainable grape seed extract, in modulating low mood and enhancing cognitive functions. The results indicate that GSEe exerts significant inhibitory effects on key neurotransmitter systems, particularly GABA-T, MAO-A, and AChE, which play crucial roles in the regulation of mood and cognition.

Regarding neurotransmitter modulation, for the first time, it is reported that GSEe inhibits GABA-T activity. This novel finding is particularly significant because GABA-T inhibition can lead to increased levels of GABA, a major inhibitory neurotransmitter in the central nervous system that is implicated in a variety of behaviors, including low mood, anxiety, stress regulation, and memory enhancement [41,42,43]. The GABA-T inhibitory effect of GSEe is likely due to its rich polyphenolic content, particularly that of OPCs. These polyphenols may interact with the enzyme’s active site or alter its conformation, thereby reducing its catalytic efficiency and preserving higher GABA levels in the synaptic cleft. By inhibiting GABA-T, GSEe could help to maintain higher levels of GABA, thus contributing to mood stabilization [44]. This possibly adds a new dimension to the beneficial potential of GSEe, highlighting its role not only in antioxidant activity but also in modulating key neurotransmitter pathways involved in mental health.

In addition to its GABA-T inhibitory activity, GSEe was found to inhibit MAO-A, an enzyme responsible for the breakdown of monoamines such as serotonin and norepinephrine. The inhibition of MAO-A can lead to increased levels of these neurotransmitters, which are crucial for mood regulation and cognitive function [45,46]. The MAO-A inhibitory effect of GSEe is likely due to the interaction of its polyphenolic constituents, particularly OPCs, with the enzyme’s flavin adenine dinucleotide (FAD) cofactor, which is essential for its catalytic activity. By binding to the FAD site, these polyphenols may reduce the enzyme’s ability to degrade monoamines, thereby increasing the availability of serotonin and norepinephrine. This finding aligns with previous research suggesting that polyphenolic compounds, such as those found in grape seed extract, possess MAO-A inhibitory properties [47]. The dual inhibition of GABA-T and MAO-A by GSEe underscores its potential as a multifaceted natural support for mood modulation. The binding of GSEe to the GABA-A receptor further supports its role in enhancing inhibitory neurotransmission [48,49].

Further, on cognitive enhancement, the inhibition of the AChE enzyme by GSEe suggests potential benefits for cognitive function, particularly in enhancing cholinergic transmission [50]. The AChE inhibitory effect of GSEe is likely due to its high polyphenolic content, particularly that of OPCs, which are known to interact with the enzyme’s active site. These polyphenols may bind to the catalytic triad of AChE, preventing the breakdown of acetylcholine and increasing its availability in the synaptic cleft. This mechanism is similar to that of several cognitive enhancers used in brain health, the function of which is related to an increase in acetylcholine availability in the brain [50,51]. Acetylcholine is essential for learning and memory processes, and its increased levels could mitigate cognitive decline [51].

The neuroprotective effects of GSEe were evident in the SH-SY5Y cell assays, where the extract demonstrated protective effects against H_2_O_2_-induced oxidative stress. The MTT and LDH assays confirmed that GSEe could mitigate cell death and maintain cellular viability under oxidative stress conditions [52]. This neuroprotection is likely due to the high antioxidant capacity of GSEe, as demonstrated by various cell-free antioxidant assays, including DPPH, ABTS, FRAP, ORAC, HORAC, and TAS [53].

Moreover, the TPC assay revealed that GSEe contains a high concentration of phenolic compounds. Phenolic compounds are known for their ability to donate hydrogen atoms and electrons, thereby neutralizing free radicals [54,55]. The high TPC in GSEe supports its strong antioxidant effects, which are crucial in mitigating oxidative damage—a key factor in the pathogenesis of neurodegenerative diseases and cognitive decline—and contribute to its overall bioactivity in modulating mood and cognitive functions [54]. The potent antioxidant activity of GSEe can be attributed to its rich content of OPCs, which are known for their ability to scavenge free radicals and reduce oxidative damage [56]. The neuroprotective effects observed in this study suggest that GSEe may help to preserve neuronal integrity and function, contributing to its cognition-enhancing properties.

The favorable safety profile of GSEe, as indicated by the lack of cytotoxicity at the tested concentrations, supports its potential as a natural support [57]. This low cytotoxicity profile is crucial for its potential mental health benefits, as it ensures that the extract does not adversely affect cell viability while exerting its positive effects [57].

These findings suggest that GSEe could be applied as a supplement targeting mood and cognitive support. Given its high polyphenolic content, particularly that of OPCs, and strong antioxidant activity, GSEe may serve as a key ingredient in natural supplements designed to improve mental well-being, reduce stress, and support cognitive function. Its eco-friendly and circular approach, as well as the use of renewable energy in its production process, further enhances its appeal as a sustainable and effective supplement ingredient.

Unlike conventional treatments for low mood and cognitive decline, which often come with adverse side effects and a risk of tolerance, GSEe offers a natural and well-tolerated alternative. Its multifaceted mechanisms of action, including neurotransmitter modulation, cognitive enhancement, neuroprotection, a high total phenolic content, and minimal cytotoxicity, make it a promising candidate for further clinical investigation.

The findings of this research are supported by two previous studies on GSEe [16,26], which provide additional context and validation for the current research. In the first study on adults aged 40–70 years with prehypertension or stage 1 hypertension, GSEe (150 and 300 mg/day for 4 months) provided a significant reduction in systolic and diastolic blood pressure, with a more pronounced effect observed in the supplement group as compared to the control group [26]. Furthermore, the same study reported improvements in the mental component summary score (MCS12) of the SF-12 health survey in the supplement group, particularly in women, which suggests GSEe’s ability to modulate mood. This complements our in vitro findings wherein GSEe demonstrated the inhibition of key neurotransmitter-regulating enzymes (GABA-T, MAO-A), suggesting a biochemical basis for its mood-enhancing effects.

Similarly, the second study, which focused on adults aged 50–75 with pre-hypertensive symptoms, also demonstrated a significant reduction in systolic and diastolic blood pressure with GSEe supplementation (300 mg/day for 16 weeks) [16]. In addition, this study provided evidence of GSEe’s anti-inflammatory and vasodilatory properties by showing reduced secretion of Inter-Cellular Adhesion Molecule-1 (sICAM) and endothelin-1 in human umbilical vein endothelial cells (HUVECs). While our study did not directly investigate inflammatory markers, the neuroprotective effects observed in SH-SY5Y cells under oxidative stress may share mechanistic similarities with the anti-inflammatory actions reported in this previous study. By reducing oxidative stress and inflammation, GSEe likely contributes to the preservation of neuronal function, further supporting its cognition-enhancing properties.

Integrating these earlier findings with our current study underscores GSEe’s multifaceted benefits, which include cardiovascular, anti-inflammatory, neuroprotective, and mood-adaptable properties. Together, these studies provide a more comprehensive understanding of GSEe’s benefits as a potential natural add-on support in cognitive and mental health.

While this study provided valuable in vitro mechanistic insights, it was limited by the absence of preclinical or clinical data. The in vitro assays, though promising, do not fully capture the complexity of mood and cognitive regulation in living organisms. Additionally, the potential for long-term use and the optimal dosage were not explored. Further validation in animal or human models is necessary to confirm the in vitro observed effects. Despite these limitations, the study offers a strong rationale by demonstrating the extract’s impact on key neurotransmitter systems and its neuroprotective, antioxidant properties, supporting its potential as a mental health supplement.

## 5. Conclusions

These findings provide evidence for GSEe’s (Enovita^®^’s) usefulness as a mood-modulating and cognition-enhancing supplement. The extract’s ability to modulate key neurotransmitter systems, protect neuronal cells from oxidative stress, exhibit strong antioxidant properties, and maintain a high total phenolic content, highlights its potential health benefits. Future studies, particularly clinical trials, are warranted to further validate these in vitro findings and to establish optimal dosing regimens for achieving the desired supporting outcomes in human subjects. Overall, the promising results from these in vitro studies lay a strong foundation for the continued exploration and development of GSEe as a viable natural supplement for enhancing mental health and cognitive function.

## Data Availability

The original contributions presented in this study are included in the article. Further inquiries can be directed to the corresponding author.
